# Metabolic Alterations Induced by a Seizure-Causing Sodium Channel Mutation and their Partial Normalization by Dietary α-Linolenic Acid in *Drosophila*

**DOI:** 10.1007/s11064-026-04673-2

**Published:** 2026-01-20

**Authors:** Karina Kruth, Junko Kasuya, Victoria Hand, Atulya Iyengar, Toshihiro Kitamoto

**Affiliations:** 1 Department of Ophthalmology and Visual Sciences, Carver College of Medicine, University of Iowa, 3107 Medical Education and Research Facility, 375 Newton Road, Iowa City, IA 52242 USA; 2 Department of Anesthesia, Carver College of Medicine, University of Iowa, 1-316 Bowen Science Building, 51 Newton Road, Iowa City, IA 52242 USA; 3 Department of Biological Sciences, University of Alabama, 2328 Science and Engineering Center, 300 Hackberry Lane, Tuscaloosa, AL 35487 USA; 4 Present Address: Department of Neuroscience and Pharmacology, Carver College of Medicine, University of Iowa, 2400 Pappajohn Biomedical Discovery Building, 169 Newton Road, Iowa City, IA 52242 USA

**Keywords:** Epilepsy, Metabolomics, Drosophila, Dietary supplementation, ϖ-3 polyunsaturated fatty acid

## Abstract

Epilepsy is increasingly recognized as a disorder with prominent metabolic disturbances, but how defined epilepsy-causing mutations reshape metabolism under controlled genetic and environmental conditions remains poorly understood. Here, we used the *Drosophila melanogaster* gain-of-function voltage-gated sodium channel (VGSC) mutant *para*^*Shu*^, a well-established model of neuronal and behavioral hyperexcitability, to characterize whole-body metabolic alterations and their modulation by dietary supplementation with the ω-3 polyunsaturated fatty acid α-linolenic acid (ALA), which strongly and specifically suppresses *para*^*Shu*^ seizure phenotypes. Adult wild-type and *para*^*Shu*^ females were reared on control or ALA-supplemented diets, and 172 metabolites were quantified using GC-MS and LC-MS. The *para*^*Shu*^ mutation induced broad metabolic alterations, including enhanced glycolysis, reduced tricarboxylic acid cycle and pentose phosphate pathway intermediates, and depletion of nicotinamide riboside and nicotinic acid adenine dinucleotide, suggesting metabolic stress, mitochondrial dysfunction, and impaired redox balance. Amino acid and nucleotide metabolism were extensively reorganized, with prominent changes in tryptophan pathways, as well as imbalances in purine and pyrimidine nucleotides and cyclic nucleotides (cAMP, cGMP). Levels of microbially derived short-chain fatty acids and indole derivatives were elevated, implicating altered gut–brain metabolic interactions. Dietary ALA partially normalized key metabolites, including succinate, 6-phosphogluconate, glycine, proline, and short-chain fatty acids, and increased N-methylnicotinamide, consistent with improved redox homeostasis and attenuated inflammation. These findings demonstrate that VGSC–driven hyperexcitability elicits coordinated metabolic and microbiota-related changes, and that ALA can mitigate these disturbances, highlighting testable metabolic targets for mechanism-based interventions in epilepsy.

**Supplementary Information:**

The online version contains supplementary material available at 10.1007/s11064-026-04673-2.

## Introduction

Epilepsy is one of the most common neurological disorders characterized by recurrent, unprovoked seizures caused by abnormal and excessive electrical activity in the brain. A variety of factors are known to contribute to the etiology of epilepsy, including brain injuries, tumors, infections, strokes, neurodegenerative diseases, and genetic mutations [1, 2]. However, the mechanisms by which these initial insults transform neuronal circuits into hyperexcitable and unstable networks remain poorly understood. In recent years, metabolomic analyses have been increasingly applied in studies of both epilepsy patients and rodent models [3–9], as metabolic disturbances are thought to be both important contributors to and key consequences of epilepsy. These studies have identified potential diagnostic and prognostic biomarkers, as well as novel therapeutic targets for epilepsy [10]. However, findings from metabolomic studies of epilepsy are often inconsistent. This is likely due in part to the heterogeneity of epilepsy etiology and in part to variability in experimental conditions (e.g., differences in genetic backgrounds, physiological states, rearing environments, sampling protocols, and analytical platforms).

The primary objective of this study was to identify changes in metabolism in a well-characterized genetic model of epilepsy, under strictly controlled genetic and environmental conditions. We utilized a powerful model organism, the fruit fly *Drosophila melanogaster*, to determine the metabolic effects of a seizure-inducing mutation in the voltage-gated sodium channel (VGSC) gene. VGSCs are essential for initiating and propagating action potentials, and they do so by responding to membrane depolarization to permit sodium ions to enter neurons [11, 12]. Among the genes implicated in seizure susceptibility, those encoding VGSCs are the most prominently associated with epilepsy disorders [1, 13]. The mammalian genome encodes nine distinct VGSC subtypes [14], and mutations in several of these—particularly *SCN1A*, *SCN2A*, and *SCN8A*—are strongly linked to specific forms of epilepsy. For example, loss-of-function mutations in *SCN1A* cause Dravet syndrome, a severe childhood epilepsy characterized by drug-resistant seizures and sudden unexpected death in epilepsy (SUDEP) [15]. *SCN1A* is predominantly expressed in GABAergic inhibitory interneurons, and its impairment results in disinhibition of excitatory circuits, enhancing neuronal excitability and promoting the synchronized firing that characterizes seizures [16]. Conversely, gain-of-function mutations in *SCN2A* and *SCN8A* lead to excessive or prolonged sodium influx in excitatory neurons, resulting in neuronal hyperexcitability and contributing to early-onset epileptic encephalopathy [17, 18].

Unlike mammals, *Drosophila* has a single VGSC gene, *paralytic* (*para*) [19, 20]. Several features make this organism a valuable platform for dissecting the roles and mechanisms of VGSCs in seizure phenotypes at the molecular, cellular, and organismal levels: the evolutionarily conserved structure and function of the VGSC genes, the simplicity of the fly nervous system, and the availability of advanced genetic tools and comprehensive genomic information. *para*^*Shu*^, originally called *Shudderer* [21], is a gain-of-function allele of *para* in which a missense mutation leads to replacement of a conserved methionine residue with isoleucine in homology domain III of the VGSC protein [22]. *para*^*Shu*^ is unique among *para* variants in that the adult mutants exhibit a range of dominant seizure-related phenotypes, including characteristic “shuddering” or spontaneous tremors, spontaneous convulsions characterized by synchronized spike discharges, increased susceptibility to electroconvulsive and heat-induced seizures, and ether-induced leg shaking [22]. Some of these *para*^*Shu*^ phenotypes closely resemble severe VGSC–related epilepsy disorders in humans. Thus, this model offers a well-defined genetic basis for studying hyperexcitability and its metabolic consequences.

Both the penetrance and expressivity of neurological phenotypes are highly sensitive to environmental factors, as neural circuits are inherently plastic in their development and function. The *para*^*Shu*^ mutant phenotype is no exception. In our previous study, we found that dietary supplementation with milk whey significantly mitigated seizure phenotypes in *para*^*Shu*^ mutants as well as in other hyperexcitable mutants [23]. Follow-up investigations identified the ω−3 polyunsaturated fatty acid α-linolenic acid (ALA; 18:3n-3) as a key dietary component underlying this diet-dependent phenotypic suppression [24]. Notably, the effects of ALA are highly specific: another polyunsaturated fatty acid, linoleic acid (LA; C18:2n-6), as well as several saturated short-, medium-, and long-chain fatty acids, had no effect. Importantly, dietary ALA suppresses hyperexcitability at a final concentration of 1 mM (approximately 0.028% w/v) [24], which is far lower than the high-fat levels used in ketogenic (high-fat, low-carbohydrate) diets to reduce seizure severity in patients with epilepsy [25]. This low effective concentration further supports the idea that ALA acts through mechanisms distinct from those underlying ketogenic diet–based seizure control. ALA is present predominantly in plant-based foods such as flaxseed and walnuts [26]. In mammals, it is an essential nutrient and a precursor for long-chain ω−3 fatty acids, including eicosapentaenoic acid (EPA; 20:5n-3) and docosahexaenoic acid (DHA; 22:6n-3) [27]. In *Drosophila*, ALA is not essential [28], but it has been implicated in diverse biological processes, including sensory perception [29] and cholesterol uptake [30]. Despite the specific and robust suppression of seizure phenotypes by dietary ALA supplementation, the mechanisms underlying these diet-dependent phenotypic modifications remain unclear. One plausible hypothesis is that ALA’s anti-inflammatory properties play a critical role, as ω−3 fatty acids are known to compete with ω−6 linoleic acid/arachidonic acid pathways, thereby shifting lipid mediator production away from pro-inflammatory prostaglandins and leukotrienes [31]. This idea is further supported by our previous finding that reduced function of glutathione S-transferase S1 (GstS1), a putative ortholog of mammalian hematopoietic prostaglandin D synthase, similarly suppresses seizure phenotypes in *para*^*Shu*^ mutants [32].

In this study, we performed metabolomic analyses using both GC-MS and LC-MS to identify metabolic changes induced by the *para*^*Shu*^ mutation and by dietary ALA supplementation. Our finding that this well-characterized seizure-causing mutation and a defined dietary intervention generate metabolic profiles distinct from those of their wild-type counterparts provides fundamental insights into the relationship between genetic susceptibility to seizures and metabolic regulation.

## Materials and Methods

### Fly Stocks, Crosses, and Culture Conditions

*Drosophila melanogaster* were reared at 25 °C and 65% humidity, on a 12-hour light/dark cycle, and on the cornmeal/glucose/yeast/agar medium that was developed by Edward Lewis [33] and modified by Rodney Williamson (Beckman Research Institute of the City of Hope, Duarte, CA). The exact composition of the diet was previously described [23]. The *Canton-S* (*CS*) strain was used as the wild-type control. The X-linked VGSC mutant strain *para*^*Shu*^ [21, 22] was obtained from Mr. Rodney Williamson (Beckman Research Institute of the Hope, CA) and backcrossed into the wild-type background for 26 generations [22]. The *para*^*Shu*^ mutation was maintained in a heterozygous state using the *FM7* X-chromosome balancer (i.e., *para*^*Shu*^/*FM7*), because *para*^*Shu*^ hemizygous males (*para*^*Shu*^/*Y*) and homozygous females (*para*^*Shu*^/*para*^*Shu*^) are extremely unfit and rarely survive or reproduce. For metabolomic analyses, *para*^*Shu*^*/FM7* females were crossed with wild-type +/*Y* males to remove the *FM7* balancer and generate heterozygous *para*^*Shu*^*/+* females. These *para*^*Shu*^/+ heterozygotes and +/+ control flies were reared on either a standard diet or a diet supplemented with 0.05% (w/v, 1.8 mM) ALA until adulthood. Virgin females (+/+ or p*ara*^*Shu*^/+) were collected within six hours of eclosion and transferred to vials containing a standard diet, with 10–20 flies per vial. Flies aged 2 days were then collected, flash-frozen in liquid nitrogen, and stored at − 80 °C for subsequent metabolomic analysis.

### Metabolomic Analysis

#### GC-MS Analysis

Metabolomic analysis was performed using GC-MS and LC-MS at the University of Iowa Metabolomics Core Facility (https://diabetes.medicine.uiowa.edu/research/metabolomics-core-facility). The in-house metabolomics library, including retention times and m/z values for target ions, is shown in Supplementary Table S1. Whole-body fly samples were lyophilized for 2 h prior to homogenization (using bead mill homogenizer) in 18:1 (µL: mg wet tissue weight) ice-cold 2:2:1 methanol/acetonitrile/water extraction buffer containing a mixture of internal standards (D4-citric acid, D4-succinic acid, D8-valine, and U13C-labeled glutamine, glutamic acid, lysine, methionine, serine, and tryptophan; Cambridge Isotope Laboratories). Homogenates were rotated for 1 h at −20 °C. Homogenates were centrifuged for 10 min at 21,000 x g, and 150 µL of the cleared metabolite extracts were transferred to autosampler vials and dried using a SpeedVac vacuum concentrator (Thermo). Dried metabolite extracts were reconstituted in 30 µL of 11.4 mg/mL methoxyamine (MOX) in anhydrous pyridine, vortexed for 5 min, and heated for 1 h at 60 °C. Next, 20 µL of N, O-Bis(trimethylsilyl)trifluoroacetamide (TMS) was added to each sample, after which samples were vortexed for 1 min and heated for 30 min at 60 °C. Derivatized samples were analyzed by GC-MS. For each derivatized sample, 1 µL was injected into a Trace 1300 GC (Thermo) fitted with a TraceGold TG-5SilMS column (Thermo) operating under the following conditions: split ratio = 20:1, split flow = 24 µL/minute, purge flow = 5 mL/minute, carrier mode = Constant Flow, and carrier flow rate = 1.2 mL/minute. The GC oven temperature gradient was as follows: 80 °C for 3 min, increasing at a rate of 20 °C/minute to 280 °C, and holding at a temperature at 280 °C for 8 min. Ion detection was performed using an ISQ 7000 mass spectrometer (Thermo) operated from 3.90 to 21.00 min in EI mode (−70 eV) using select ion monitoring (SIM).

#### LC-MS Analysis

Whole-body fly samples were lyophilized and transferred to ceramic bead tubes. For each sample, 18-fold (w/v) extraction solvent (with 9 heavy internal standards) was added and samples were homogenized. After homogenization, samples were rotated at −20 °C for 1 h and then centrifuged at 21,000 x g for 10 min. For each sample, 200 µL of the extract supernatant was transferred to microcentrifuge tubes, and the extracts were dried using a SpeedVac Concentrator. Dried extracts were reconstituted in 20 µL acetonitrile/water (1:1 v/v) and vortexed well, and then kept at −20 °C overnight. The following day, samples were centrifuged and the supernatant was transferred to LC-MS autosampler vials for analysis. LC-MS data were acquired on a Thermo Q Exactive hybrid quadrupole Orbitrap mass spectrometer with a Vanquish Flex UHPLC system or Vanquish Horizon UHPLC system. The LC column used was a Millipore SeQuant ZIC-pHILIC (2.1 × 150 mm, 5 μm particle size) with a ZIC-pHILIC guard column (20 × 2.1 mm). The injection volume was 2 µL. The mobile phase was as follows: Solvent A (20 mM ammonium carbonate [(NH_4_)_2_CO_3_] and 0.1% ammonium hydroxide (v/v) [NH_4_OH]) and Solvent B (acetonitrile). The flow rate was 0.150 mL/min. The gradient started at 80% B, decreased to 20% B over 20 min, returned to 80% B in 0.5 min, and was held at 80% for 7 min [34]. The mass spectrometer was operated in full-scan, polarity-switching mode from 1 to 20 min, with the spray voltage set to 3.0 kV, the heated capillary held at 275 °C, and the HESI probe held at 350 °C. The sheath gas flow was set to 40 units, the auxiliary gas flow to 15 units, and the sweep gas flow to 1 unit. MS data acquisition was performed across a range of m/z 70–1,000, with the resolution set at 70,000, the AGC target at 1 × 106, and the maximum injection time at 200 ms [34].

#### Data Processing and Statistical Analysis

Raw data were analyzed using TraceFinder 5.1 (Thermo). Metabolite identification and annotation required at least two ions (target + confirming), a unique retention time specific to the ions, and the retention time of a reference standard previously determined in-house. A pooled-sample generated prior to derivatization was analyzed at the beginning of the analytical run, at a set interval during the analytical run, and at the end the analytical run, to correct peak intensities using the NOREVA tool [35]. NOREVA-corrected data were then normalized to the sum total of signal per sample to control for extraction, derivatization, and/or loading effects. For short-chain fatty acids (SCFAs), acquired LC-MS data were processed using the Thermo Scientific TraceFinder 4.1 software, and metabolites were identified using the University of Iowa Metabolomics Core facility standard-confirmed, in-house library. Analyte signal was corrected by normalizing to the deuterated analyte signal, and the signal obtained from a processing blank (PB) was subtracted. The processed data were evaluated using functional modules for statistical analysis in MetaboAnalyst 5.0 [36], as well as GraphPad Prism 10 (GraphPad Software, Inc., Boston, MA).

## Results

### Alterations in Overall Whole-Body Metabolism Induced by *para*^*Shu*^ and Dietary Supplementation with ALA

**Fig. 1:**
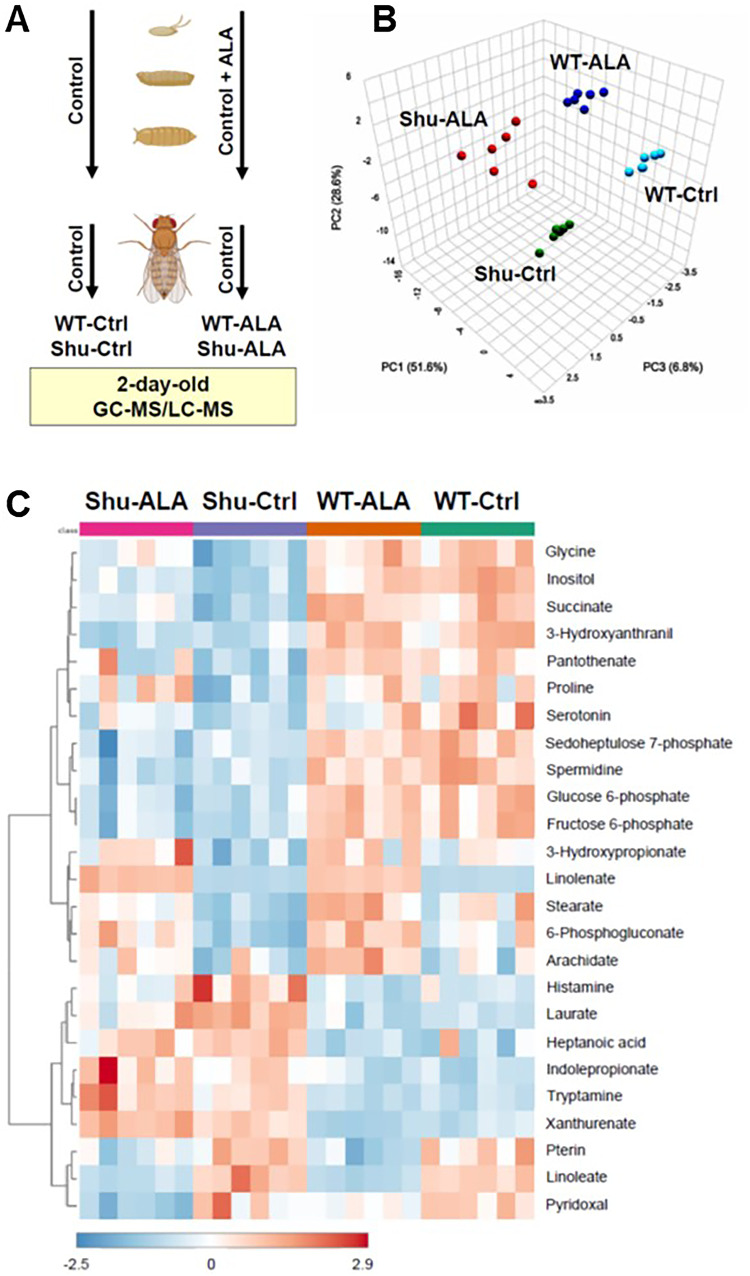
Whole-body metabolomic analysis of control and *para*^*Shu*^ mutant flies with and without ALA treatment. **A** Schematic of the experimental design. WT-Ctrl and Shu-Ctrl flies were maintained on a control diet throughout the experiment. WT-ALA and Shu-ALA flies were reared on the ALA diet until eclosion, then transferred to a control diet for two days before analysis. Each group included six biological replicates, and each replicate included approximately 50 flies. **B** Three-dimensional principal component scatterplot. **C** Heatmap of hierarchical clustering based on the most significantly altered metabolites

To identify metabolic changes resulting from the *para*^*Shu*^ mutation and dietary ALA supplementation, we performed whole-body metabolomic analysis on adult female *Drosophila* using GC-MS and LC-MS (Fig. 1A). Four experimental groups were analyzed: (1) wild-type *Canton-S* (*CS*) flies on a control diet (WT-Ctrl), (2) *para*^*Shu*^ heterozygotes on a control diet (Shu-Ctrl), (3) *CS* flies on a diet supplemented with ALA (WT-ALA), and (4) *para*^*Shu*^ heterozygotes on a diet supplemented with ALA (Shu-ALA). A total of 172 metabolites were analyzed (Supplementary Table S2). Principal component analysis (PCA) showed that for each group the six biological replicates were tightly clustered and that the four groups were clearly separated (Fig. 1B). Thus, both the *para*^*Shu*^ mutation and ALA supplementation induce reproducible changes in whole-body metabolite profiles and these changes are distinct.

One-way ANOVA was used to compare metabolite levels across the four experimental groups (Supplementary Table S3). Heatmaps of the 25 most significantly altered metabolites highlight the primary effects of the *para*^*Shu*^ mutation and ALA treatment on the metabolome (Fig. 1C): seven of the affected metabolites are directly involved in fatty acid metabolism (linolenate, linoleate, laurate, stearate, heptanoic acid, arachidate, and 3-hydroxypropionate); five are associated with tryptophan metabolism (tryptamine, xanthurenate, 3-hydroxyanthranilic acid, serotonin, and indolepropionate); and four are involved in glycolysis or the pentose phosphate pathway (PPP; fructose 6-phosphate, glucose 6-phosphate, 6-phosphogluconate, and sedoheptulose 7-phosphate). Notably, significant effects were observed not only for host-derived metabolic products but also for metabolites of microbial origin, such as indolepropionate.

Given that the primary goal of this study was to identify metabolic changes associated with the hyperexcitability phenotype caused by the *para*^*Shu*^ mutation, as well as with the suppression of *para*^*Shu*^ phenotypes by dietary ALA, we performed two pairwise comparisons: (1) Shu-Ctrl vs. WT-Ctrl and (2) Shu-ALA vs. Shu-Ctrl. These analyses were conducted using Student’s t-tests with a false discovery rate (FDR) threshold of < 0.05. The number of significantly altered metabolites was 55 for Shu-Ctrl vs. WT-Ctrl, and 11 for Shu-ALA vs. Shu-Ctrl (Table 1). Seven metabolites were common to both comparisons (indicated in bold in Table 1). Notably, the levels of all these metabolites changed in opposite directions in response to the *para*^*Shu*^ mutation and ALA treatment, consistent with ALA having suppressive effects on the physiological and behavioral phenotypes of *para*^*Shu*^.

**Table 1:** List of metabolites significantly affected by the *para*^*Shu*^ mutation (A) and by dietary ALA supplementation (B)

A		
Shu-Ctrl/WT-Ctrl		
Metabolite	FC	FDR
Laurate	1.7032	1.8131e-05
**Propionic acid**	**2.652**	**4.4605e-05**
Inositol	0.75552	6.5209e-05
Xanthurenate*	1.4362	0.00026273
cGMP	2.4317	0.00041612
**N-methylnicotinamide**	**0.50196**	**0.00041612**
Arginine*	0.79643	0.00041612
Tryptamine*	1.7373	0.00088473
Nudifloramide	0.66979	0.00088473
Sedoheptulose 7-phosphate	0.69263	0.00088473
**Succinate**	**0.76745**	**0.00088473**
GMP	1.2756	0.00088473
Spermidine	0.83216	0.00088473
Pyruvate	1.4484	0.0010302
Glycine*	**0.75713**	**0.0010302**
CMP	1.5438	0.0013928
Nicotinamide riboside	0.52942	0.0017985
dAMP	1.6643	0.0024425
Fructose 6-phosphate	0.68543	0.0027042
Pantothenate	0.78695	0.0031457
Alpha-Ketoglutarate	1.4932	0.003699
Nicotinic acid adenine dinucleotide	0.70015	0.003699
3-Hydroxyanthranilic acid*	0.78518	0.003699
Serotonin*	0.86251	0.003699
UMP	1.4767	0.0041324
Glucose 6-phosphate	0.66811	0.0051104
Pimelate	0.79427	0.0051652
Indolepropionate*	1.1947	0.0051652
Thymidine	1.758	0.0081491
dGDP	0.77616	0.008532
XMP	0.46705	0.012932
CDP	1.8856	0.012932
Lactate	1.3062	0.016151
Beta-Alanine*	0.83125	0.016771
Histamine*	2.0081	0.016984
Butyric acid	1.2829	0.017975
Fumarate	0.48729	0.018805
Tryptophol*	0.81233	0.018805
**6-Phosphogluconate**	**0.82089**	**0.018805**
Gamma-aminobutyrate*	1.2513	0.018816
Proline*	**0.81779**	**0.019153**
3-Hydroxykynurenine*	0.76602	0.023844
O-Phosphoethanolamine	1.3542	0.024609
Homocysteine*	1.358	0.025338
Cadaverine*	0.85495	0.025865
Pentadecanoate	0.8513	0.026452
Xanthosine	0.61799	0.027825
**Stearate**	**0.77327**	**0.027825**
Alpha-Ketoisovalerate*	1.2748	0.027877
Thymine	1.2917	0.029036
3-Hydroxypropionate	0.82353	0.030201
Heptanoic acid	1.3747	0.032302
dCMP	1.1582	0.043924
Glutamine*	0.88733	0.045465
Alanine*	0.9082	0.046378

B		
Shu-ALA/Shu-Ctrl		
Metabolite	FC	FDR
Linolenate	12.225	1.4808e-08
Linoleate	0.48097	0.0013089
**Stearate**	**1.2795**	**0.0019819**
**6-Phosphogluconate**	**1.3278**	**0.0042623**
**Propionic acid**	**0.65447**	**0.0078401**
Pyridoxal	0.51448	0.028726
**N-methylnicotinamide**	**1.5298**	**0.031009**
**Proline**	**1.2443**	**0.049725**
**Glycine**	**1.184**	**0.049725**
**Succinate**	**1.1469**	**0.049725**
Pterin	0.87857	0.049725

*Metabolites directly involved in amino acid metabolism. Metabolites shown in bold indicate those affected by both the *para*^*Shu*^ mutation and ALA supplementation

**Fig. 2:**
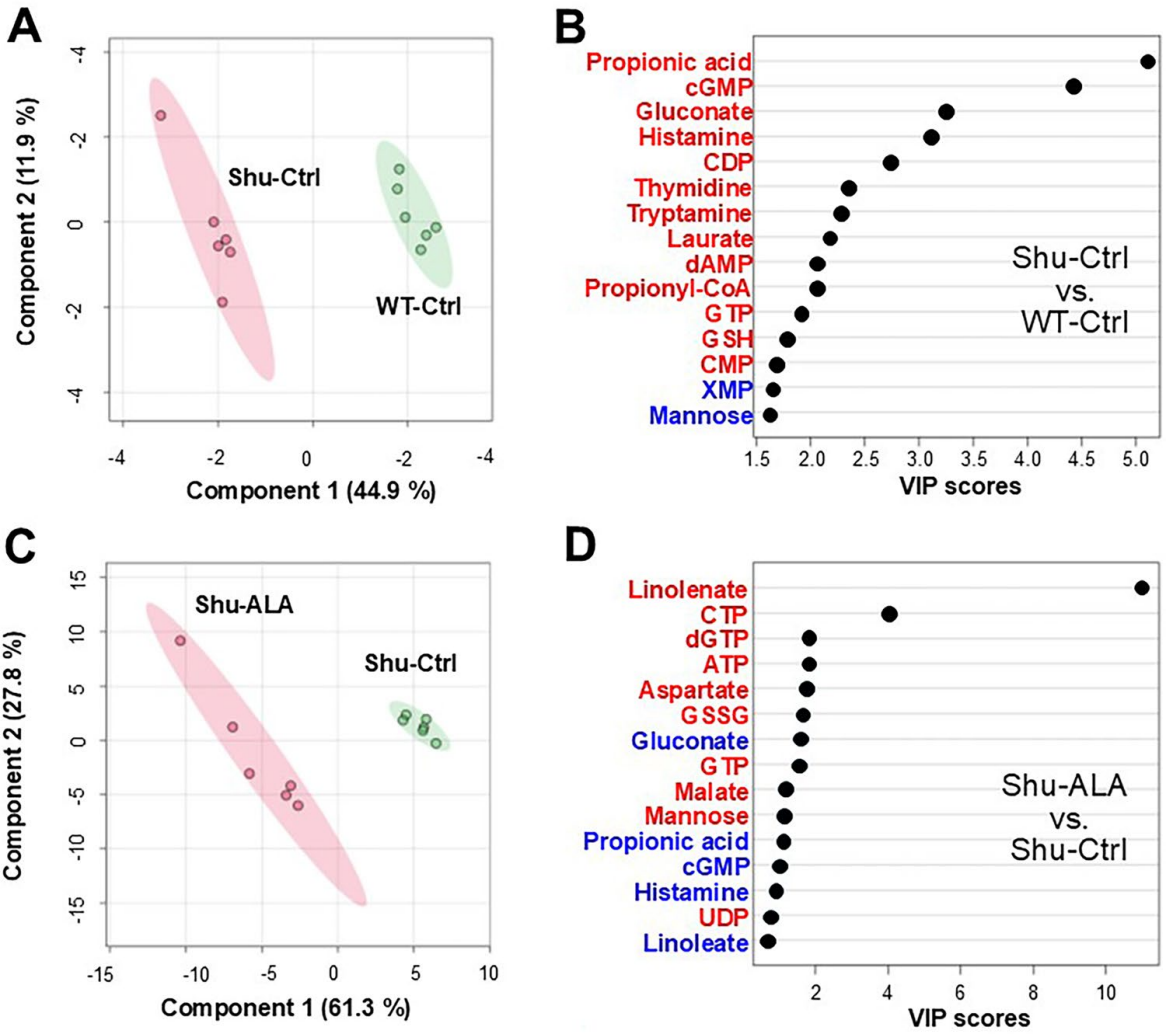
Multivariate analysis of metabolic effects of the *para*^*Shu*^ mutation and dietary ALA supplementation. **A**, **C** Partial least squares discriminant analysis (PLS-DA) score plots comparing Shu-Ctrl and WT-Ctrl flies **A**, and Shu-ALA and Shu-Ctrl flies **C**. **B**, **D** Variable importance in projection (VIP) plots showing the metabolites that contributed most to the group separation observed in **A** and **C**. Metabolites in red were elevated and those in blue were reduced in Shu-Ctrl compared with WT-Ctrl **B**, and in Shu-ALA compared with Shu-Ctrl **D**

Group separation was visualized using Partial Least Squares Discriminant Analysis (PLS-DA; Fig. 2A, C), and the metabolites most responsible for this distinction were identified based on Variable Importance in Projection (VIP) scores (Fig. 2B, D). A VIP score greater than 1.0 is generally considered important for group discrimination. In the comparison between Shu-Ctrl and WT-Ctrl, propionic acid, cGMP, gluconate, and histamine had high VIP scores (5.1, 4.4, 3.2, and 3.1, respectively) indicative of strong contributions to group separation (Fig. 2C). In the comparison between Shu-Ctrl and Shu-ALA, these metabolites likewise had high VIP scores (1.1, 0.99, 1.53, and 0.94, respectively; Fig. 2D), and thus also appear to contribute to separation. Notably, *para*^*Shu*^ and ALA had opposite effects on these metabolites. In the *para*^*Shu*^ mutant the metabolite levels increased whereas in the context of dietary ALA supplementation they decreased (Fig. 2C, D).

**Fig. 3:**
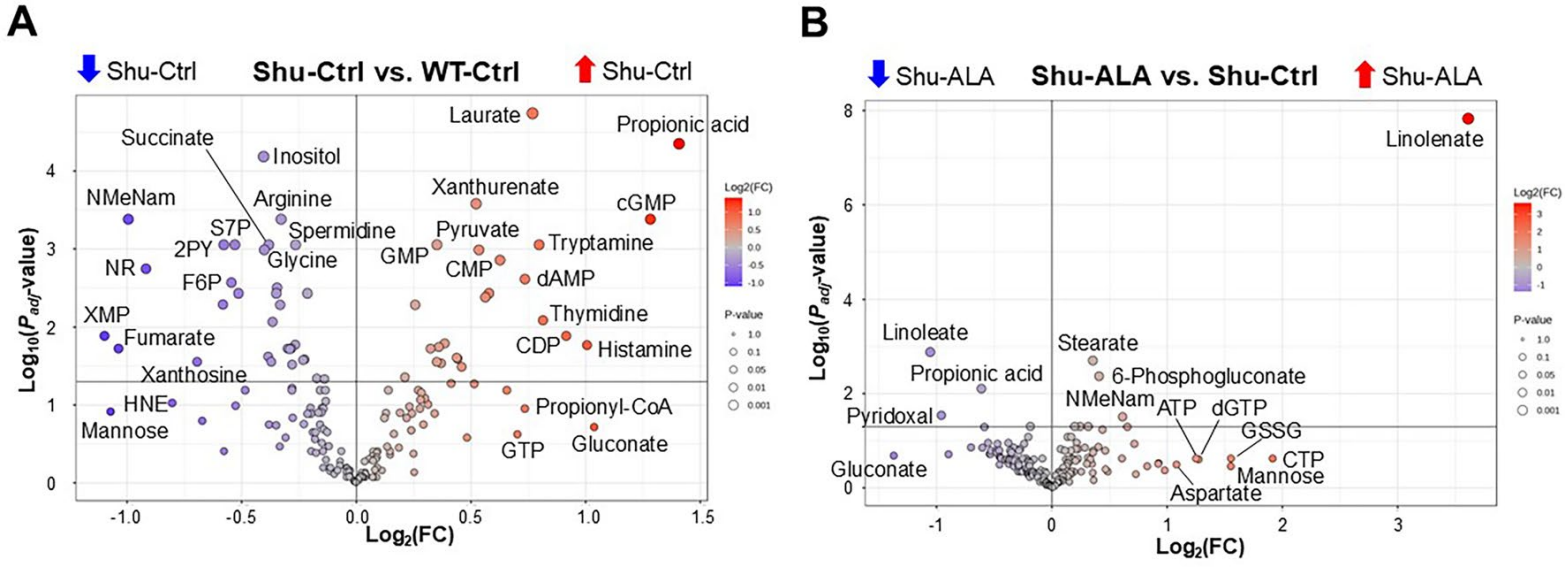
Volcano plots of metabolite changes induced by the *para*^*Shu*^ mutation and ALA supplementation. **A** Shu-Ctrl vs. WT-Ctrl flies. **B** Shu-ALA vs. Shu-Ctrl flies. Adjusted *P*-value (*P*_*adj*_) and fold change (FC) are indicated. Abbreviations of the labeled metabolites: *NMeNam* N-methylnicotinamide, *S7P* sedoheptulose 7-phosphate, *2PY* N-methyl-2-pyridone-5-carboxamide, *NR* nicotinamide riboside, *HNE* 4-hydroxy-2-nonenal, *XMP* xanthosine monophosphate, *F6P* fructose-6-phosphate, *GSSG* glutathione disulfide

Metabolites that are significantly up- or downregulated by the *para*^*Shu*^ mutation (Fig. 3A) or dietary ALA supplementation (Fig. 3B) were shown using volcano plots. Major metabolic pathways affected by these conditions were identified by pathway analysis using the MetaboAnalyst tool [36]. Applying cutoff criteria of FDR < 0.01 and a pathway impact score (PIS) > 0.3, we identified 18 significantly affected metabolic pathways in the Shu-Ctrl vs. WT-Ctrl comparison (Table 2). Among these, the six pathways with the highest PIS values were: (1) Alanine, aspartate, and glutamate metabolism (PIS = 0.804, FDR = 0.000106); (2) Nicotinate and nicotinamide metabolism (PIS = 0.773, FDR = 0.000593); (3) Pentose phosphate pathway (PPP) (PIS = 0.722, FDR = 8.98 × 10⁻⁵); (4) Glycine, serine, and threonine metabolism (PIS = 0.667, FDR = 0.00078); (5) Arginine biosynthesis (PIS = 0.629, FDR = 0.00326); and (6) Tryptophan metabolism (PIS = 0.549, FDR = 0.000459). In contrast, no pathways met these stringent criteria in the Shu-ALA vs. Shu-Ctrl comparison. However, when more relaxed cutoffs were applied (FDR < 0.05 and PIS > 0.3), Vitamin B6 metabolism (FDR = 0.026, PIS = 0.33) was found to be significantly affected by ALA in this comparison. In the following sections, we highlight key effects on metabolites within the pathways that are most significantly influenced by the *para*^*Shu*^ mutation or dietary ALA treatment.

**Table 2:** Metabolic pathways significantly altered by the *para*^*Shu*^ mutation

	Total Compound	Hits	FDR	Impact
Alanine, aspartate and glutamate metabolism	23	10	0.00010605	0.80406
Nicotinate and nicotinamide metabolism	9	7	0.00059349	0.77357
Pentose phosphate pathway	22	9	8.9776e-05	0.72251
Glycine, serine and threonine metabolism	29	6	0.00078037	0.66696
Arginine biosynthesis	12	9	0.0032563	0.62857
Tryptophan metabolism	30	8	0.00045934	0.54863
Pyrimidine metabolism	40	15	0.0032563	0.52349
Glutathione metabolism	26	9	0.0066485	0.51293
Arginine and proline metabolism	29	6	0.00045934	0.51111
Purine metabolism	68	22	0.00010605	0.48849
Citrate cycle (TCA cycle)	20	10	0.00059349	0.43363
Pyruvate metabolism	23	7	0.0012616	0.46638
Starch and sucrose metabolism	14	4	0.0016183	0.41616
Glycolysis/Gluconeogenesis	26	8	0.0029461	0.40948
Histidine metabolism	9	2	0.007751	0.4
Tyrosine metabolism	33	5	0.00078037	0.38612
Cysteine and methionine metabolism	32	5	0.00073611	0.34704
Propanoate metabolism	20	7	8.9776e-05	0.33682

### Central Carbon Metabolism

**Fig. 4:**
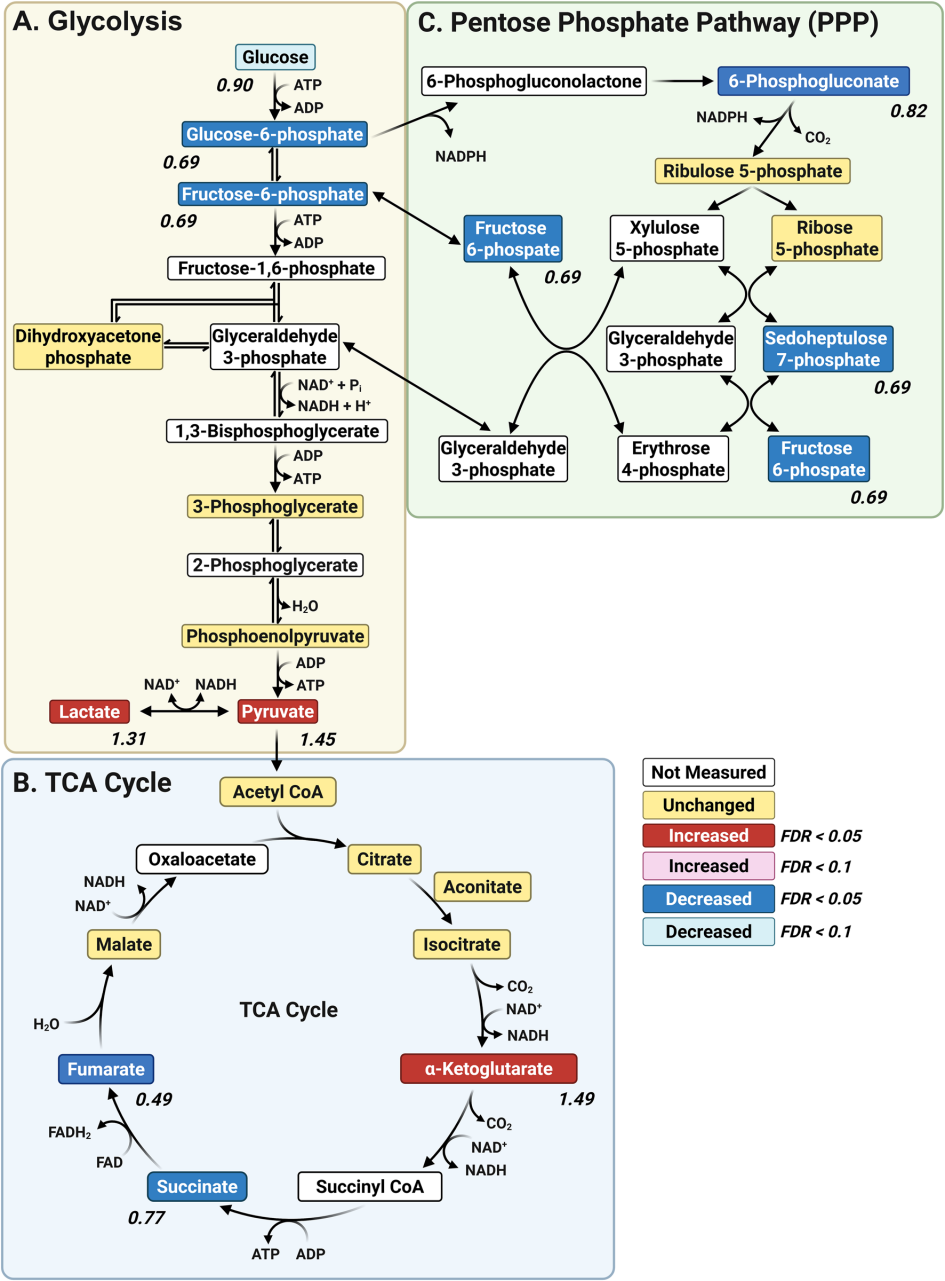
Effects of the *para*^*Shu*^ mutation on central carbon metabolism. Shown are metabolites of central carbon metabolism, including those involved in glycolysis **A**, the tricarboxylic acid (TCA) cycle **B**, and the pentose phosphate pathway **C**. Differences in metabolites in Shu-Ctrl relative to WT-Ctrl flies are indicated as follows: red boxes, upregulated (FDR < 0.05); pink boxes, upregulated (FDR < 0.1); blue boxes, downregulated (FDR < 0.05); cyan boxes, downregulated (FDR < 0.1); yellow boxes, unchanged; white boxes, not measured. Fold change (FC) values are shown, where FC > 1 denotes an increase and FC < 1 denotes a decrease in metabolite levels in Shu-Ctrl flies relative to WT-Ctrl flies

Central carbon metabolism (Fig. 4) is a network of biochemical pathways that processes carbon sources to generate energy, biosynthetic precursors, and reducing equivalents. The major central carbon mechanism pathways include glycolysis, the tricarboxylic acid (TCA) cycle, and the pentose phosphate pathway (PPP) [37], all of which were significantly affected in *para*^*Shu*^ mutants (Table 2). The primary end product of glycolysis is pyruvate, and under anaerobic conditions it is converted to lactate through fermentation; this reaction regenerates nicotinamide adenine dinucleotide (NAD⁺) to allow glycolysis to continue (Fig. 4A). Levels of both pyruvate (FC = 1.44, FDR = 0.001) and lactate (FC = 1.30, FDR = 0.016) were significantly higher in *para*^*Shu*^ mutants than wild-type flies, indicating that glycolytic flux is high in the mutants. However, levels of upstream intermediates of glycolysis, such as glucose 6-phosphate (FC = 0.66, FDR = 0.0051) and fructose 6-phosphate (FC = 0.68, FDR = 0.0027), were lower. This could potentially be due to increased consumption downstream, which would lead to substrate depletion. It is also possible that alternative sources of pyruvate are utilized, for example the three-carbon amino acids alanine, serine, and cysteine. Within the TCA cycle (Fig. 4B), levels of succinate (FC = 0.76, FDR = 0.00088) and fumarate (FC = 0.48, FDR = 0.018) were lower in the *para*^*Shu*^ mutants, suggesting that the pathway is disrupted at the middle or late stage. Conversely, α-ketoglutarate was elevated in the mutant (FC = 1.49, FDR = 0.0037); this could reflect either a reduction in the conversion of α-ketoglutarate to downstream TCA intermediates or an increase in glutaminolysis (with glutamine metabolized to glutamate and subsequently to α-ketoglutarate). Collectively, these changes suggest that flux of the TCA cycle is impaired. The *para*^*Shu*^ mutants also had significantly lower levels of 6-phosphogluconate (FC = 0.82, FDR = 0.018) and sedoheptulose 7-phosphate (FC = 0.69, FDR = 0.00088), both of which are PPP intermediates, as well as fructose 6-phosphate (FC = 0.68, FDR = 0.0027), a metabolite that is both a glycolytic intermediate and a product of the non-oxidative branch of the PPP (Fig. 4C).

Treatment of *para*^*Shu*^ mutants with ALA partially reversed some of the effects of *para*^*Shu*^ on the central carbon metabolism. Dietary ALA supplementation led to increased levels of succinate (FC = 1.14, FDR = 0.049) and 6-phosphogluconate (FC = 1.32, FDR = 0.0042), suggesting that function of the TCA cycle and PPP were improved. However, it did not lead to significant increases in levels of core glycolytic intermediates (FDR < 0.05), suggesting that that ALA does not substantially affect glycolysis.

### Amino Acid Metabolism

The *para*^*Shu*^ mutation profoundly alters amino acid metabolism. Of the 55 metabolites that differed significantly (FDR < 0.05) between *para*^*Shu*^ and wild-type flies, 18 are directly involved in amino acid metabolism (marked with an asterisk in Table 1A). Among them, those that were present at significantly higher levels in *para*^*Shu*^ flies were the neurotransmitter-related metabolites histamine (FC = 2.0, FDR = 0.017) and gamma-aminobutyrate (GABA; FC = 1.25, FDR = 0.019). Another was the methionine derivative homocysteine (FC = 1.35, FDR = 0.025). Another set of amino acids and their derivatives were lower in *para*^*Shu*^ mutants. These included alanine (FC = 0.90, FDR = 0.046), beta-alanine (FC = 0.83, FDR = 0.016), glutamine (FC = 0.88, FDR = 0.045), glycine (FC = 0.75, FDR = 0.001), proline (FC = 0.81, FDR = 0.019), arginine (FC = 0.79, FDR = 0.0004), and cadaverine, a lysine derivative (FC = 0.85, FDR = 0.026).

**Fig. 5:**
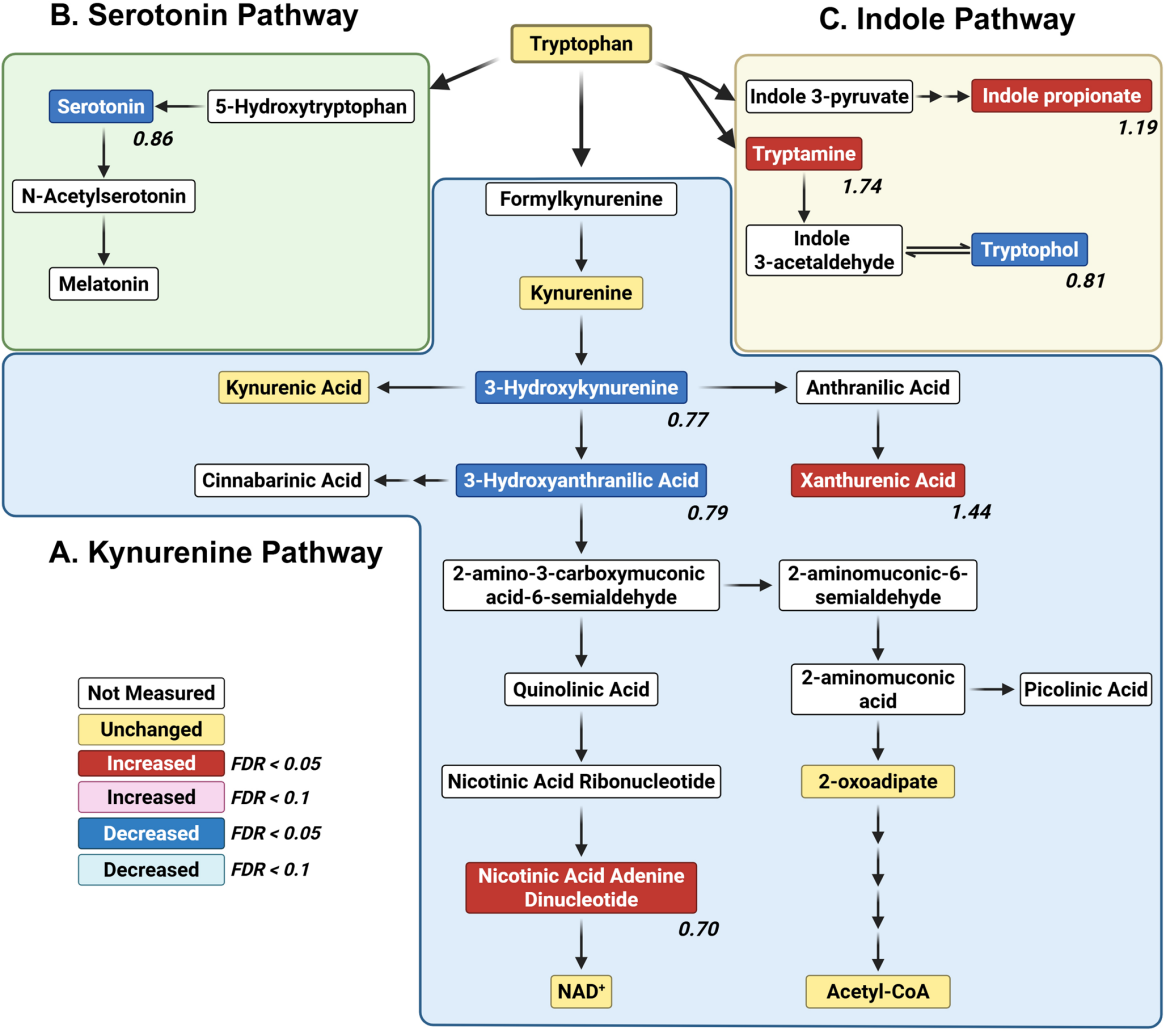
Effects of the *para*^*Shu*^ mutation on metabolites in the tryptophan pathway. Tryptophan metabolites in the kynurenine **A**, serotonin **B**, and indole **C** pathways are shown. Differences between Shu-Ctrl and WT-Ctrl flies are indicated as follows: Red boxes, upregulated (FDR < 0.05); pink boxes, upregulated (FDR < 0.1); blue boxes, downregulated (FDR < 0.05); cyan boxes, downregulated (FDR < 0.1); yellow boxes, unchanged; white boxes, not measured. Fold change (FC) values are shown, where FC > 1 denotes an increase and FC < 1 denotes a decrease in metabolite levels in Shu-Ctrl flies relative to WT-Ctrl flies

Beyond its role in protein synthesis, tryptophan is a precursor to several important bioactive compounds, including NAD⁺, which supports energy metabolism and redox homeostasis [38]), and serotonin, which functions as both a neurotransmitter and a neuromodulator. Tryptophan is metabolized through three major pathways—the kynurenine, serotonin, and indole pathways [39–41]. All of these are markedly affected by the *para*^*Shu*^ mutation (Fig. 5). In the kynurenine pathway (Fig. 5A), levels of xanthurenate (FC = 1.44, FDR = 0.00026) were higher, whereas those of 3-hydroxykynurenine (FC = 0.76, FDR = 0.023) and 3-hydroxyanthranilic acid (FC = 0.78, FDR = 0.0037) were lower. These changes suggest that the metabolic flux through branches of the kynurenine pathway that support NAD⁺ synthesis may be impaired in *para*^*Shu*^ mutants, potentially disrupting redox regulation. In contrast, metabolites within the oxoadipate-acetyl-CoA branch of tryptophan degradation were not significantly altered in *para*^*Shu*^ mutants. In the serotonin pathway (Fig. 5B), serotonin levels were lower (FC = 0.86, FDR = 0.036), indicating that serotonergic signaling was diminished. In the indole pathway (Fig. 5C), levels of tryptamine and indolepropionate were higher (tryptamine: FC = 1.74, FDR = 0.0088; indolepropionate: FC = 1.19, FDR = 0.0051), whereas levels of tryptophol were lower (FC = 0.81, FDR = 0.019). Notably, indolepropionate and tryptophol are not synthesized de novo by *Drosophila melanogaster*; their presence in flies therefore primarily reflects gut bacterial metabolism of tryptophan. Although tryptamine can, in principle, be synthesized by the host via aromatic L-amino acid decarboxylase, this reaction is kinetically unfavorable [42]. Thus, tryptamine detected in flies is likely derived from dietary sources and/or produced by the gut microbiota.

The effects of the *para*^*Shu*^ mutation on amino acids were partially reduced by ALA supplementation. Notably, although levels of proline and glycine—which are central to redox balance and neurotransmission—were lower in *para*^*Shu*^ mutants, they were normalized in the context of ALA supplementation (proline: FC = 1.24, FDR = 0.049; glycine: FC = 1.18, FDR = 0.049), showing that dietary ALA helps restore neurochemical and metabolic homeostasis.

### Nucleotide Metabolism

**Fig. 6:**
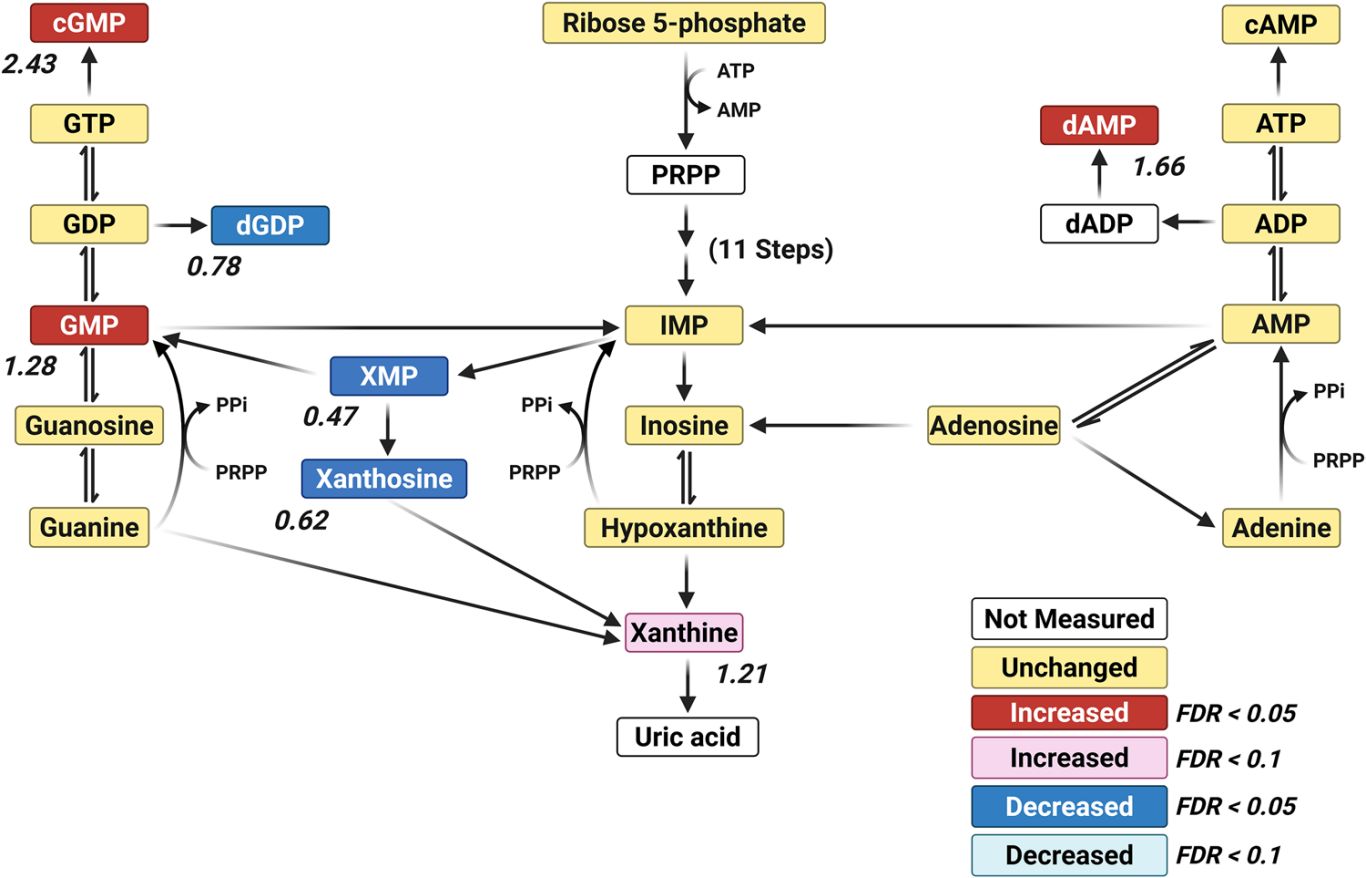
Effects of the *para*^*Shu*^ mutation on metabolites in the purine pathway. Purine metabolites are shown. Differences between Shu-Ctrl and WT-Ctrl flies are indicated as follows: red boxes, upregulated (FDR < 0.05); pink boxes, upregulated (FDR < 0.1); blue boxes, downregulated (FDR < 0.05); cyan boxes, downregulated (FDR < 0.1); yellow boxes, unchanged; white boxes, not measured. Fold change (FC) values are shown, where FC > 1 denotes an increase and FC < 1 denotes a decrease in metabolite levels in Shu-Ctrl flies relative to WT-Ctrl flies. Red boxes, upregulated (FDR < 0.05); pink boxes, upregulated (FDR < 0.1); blue boxes, downregulated (FDR < 0.05); cyan boxes, downregulated (FDR < 0.1); yellow boxes, unchanged; white boxes, not measured. Fold change (FC) values are shown, where FC > 1 denotes an increase and FC < 1 denotes a decrease in metabolite levels in Shu-Ctrl flies relative to WT-Ctrl flies

The metabolomic profile of the *para*^*Shu*^ mutant also revealed widespread perturbations in nucleotide metabolism, including both the purine and pyrimidine pathways. Levels of several pyrimidine nucleotides were higher. This was the case for CMP (FC = 1.54, FDR = 0.00139), UMP (FC = 1.48, FDR = 0.00413), CDP (FC = 1.89, FDR = 0.0129), and dCMP (FC = 1.16, FDR = 0.0439). Similar trends were observed for the nucleoside thymidine (FC = 1.76, FDR = 0.00815) and its precursor thymine (FC = 1.29, FDR = 0.029). These findings suggest that the biosynthesis and turnover of pyrimidines were increased overall. In contrast to the observed trend for an increase in pyrimidine levels, the effects on purine metabolism were inconsistent (Fig. 6). Levels of both GMP (FC = 1.28, FDR = 0.000884) and its signaling derivative cGMP (FC = 2.4317, FDR = 0.00041612) were markedly increased, consistent with a shift toward guanine nucleotide accumulation and increased cGMP-mediated signaling. However, levels of several purine intermediates were significantly lower. These included XMP (FC = 0.47, FDR = 0.0129), xanthosine (FC = 0.62, FDR = 0.0278), and dGDP (FC = 0.776). Notably, levels of cAMP were higher in the mutant (FC = 1.66, FDR = 0.00244). The contrast between this observation and the decrease in dGDP levels highlights that the deoxynucleotide balance is disrupted.

### Fatty Acid Metabolism

**Fig. 7:**
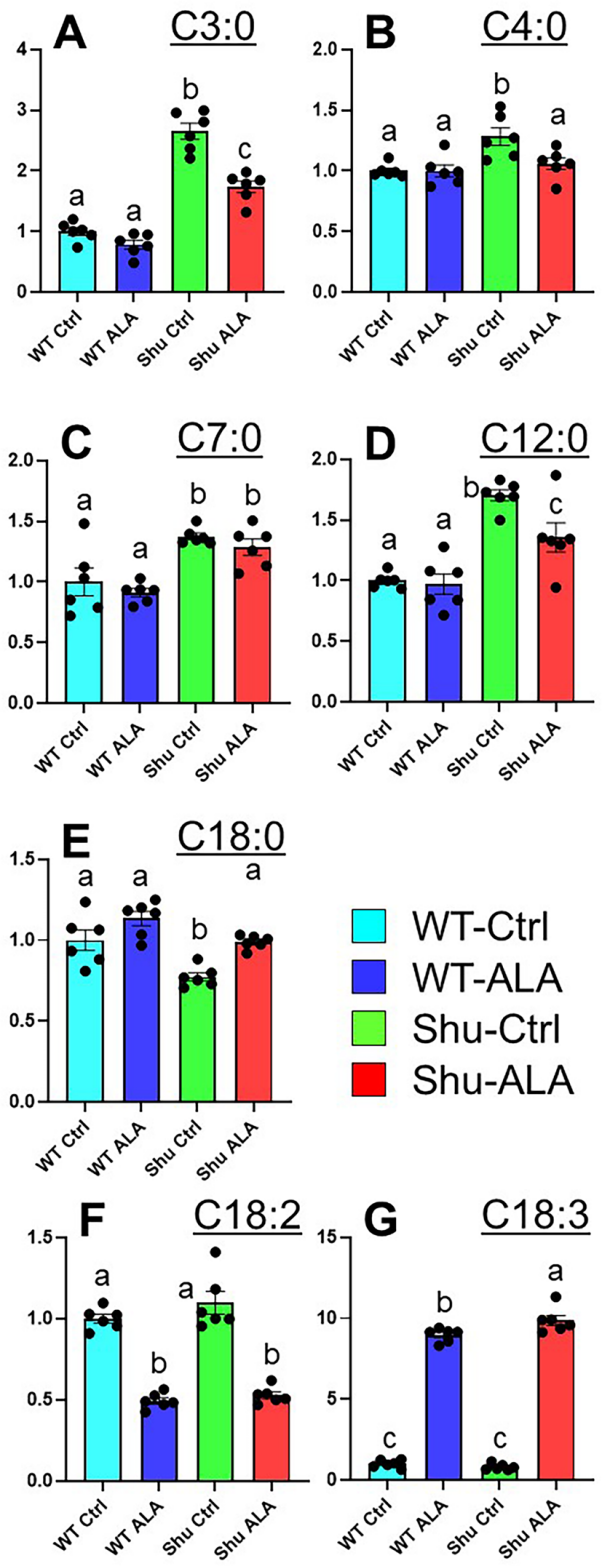
Effects of the *para*^*Shu*^ mutation and dietary ALA supplementation on levels of fatty acids. Relative levels of fatty acids in WT-Ctrl (cyan), WT-ALA (navy), Shu-Ctrl (green), and Shu-ALA (red) flies, shown as mean ± SEM for six biological replicates. **A** Propionic acid (C3:0), **B** Butyric acid (C4:0), **C** Heptanoic acid (C7:0), **D** Lauric acid (C12:0), **E** Stearic acid (C18:0), **F** LA (C18:2), **G** ALA (C18:3). Different lowercase letters (a, b, c) above bars indicate statistically significant differences among groups (groups that share a letter are not significantly different). *C3:0* propionic acid, *C4:0* butyric acid, *C7:0* heptanoic acid, *C12:0* lauric acid, *C18:0* stearic acid, *C18:2* linoleic acid, C18:3, α-linolenic acid

The *para*^*Shu*^ mutation and ALA supplementation also noticeably influenced fatty-acid metabolism. Among the saturated fatty acids, five were markedly affected. Specifically, levels of two SCFAs, propionic acid (C3:0) (FC = 2.65, FDR = 4.46 × 10^− 5^) and butyric acid (C4:0) (FC = 1.28, FDR = 0.0179), and two medium-chain fatty acids (MCFAs), heptanoic acid (C7:0) (FC = 1.37, FDR = 0.0323) and lauric acid (C12:0) (FC = 1.31, FDR = 0.0162), were markedly higher in *para*^*Shu*^ mutants (Fig. 7A–D). In contrast, levels of the long-chain fatty acid (LCFA) stearic acid (C18:0) (FC = 0.773, FDR = 0.0278) were lower (Fig. 7E). ALA treatment partially reversed most of these effects in *para*^*Shu*^ mutants. Levels of propionic acid and butyric acid were 35% and 11% lower, respectively, in ALA-treated *para*^*Shu*^ mutants (Fig. 7A, B). Levels of lauric acid and stearic acid in ALA-treated *para*^*Shu*^ mutants were also approaching to those in wild-type flies (Fig. 7D, E). The exception was heptanoic acid, which remained elevated in *para*^*Shu*^ mutants compared with wild-type flies, regardless of diet type (Fig. 7C). Among the fatty acids altered in *para*^*Shu*^ mutants, the SCFAs propionic acid and butyric acid are primarily produced by gut microbiota [43], whereas the medium- and long-chain fatty acids reflect host lipid metabolism or dietary intake and are not considered direct microbial products.

We also examined effects on linolenate and linoleate, the ionic forms of ω−3 ALA and ω−6 linoleic acid (LA), respectively. As expected, linolenate levels were significantly higher after dietary ALA supplementation in both wild-type flies (FC = 8.96, FDR = 3.67 × 10^− 10^) and *para*^*Shu*^ mutants (FC = 12.2, FDR = 1.48 × 10^− 8^ (Fig. 7G). Notably, although linolenate and linoleate cannot be interconverted enzymatically or chemically in the body, linoleate levels were approximately 50% lower in both wild-type flies and the mutants fed the ALA-supplemented diet (Fig. 7F). These findings suggest that the ω−3 and ω−6 fatty acid metabolic pathways strongly regulate one another.

## Discussion

Here, we investigated how a defined seizure-causing gain-of-function mutation in the VGSC gene alters metabolism and how these metabolic changes are modulated by dietary ALA, a potent suppressor of *para*^*Shu*^ seizure phenotypes. Our whole-body metabolomic analyses reveal that *para*^*Shu*^-driven neuronal hyperexcitability is associated with coordinated disruptions in central carbon metabolism, amino acid and nucleotide pathways, redox homeostasis, and microbiota-derived metabolites. Dietary ALA supplementation partially reversed several of these alterations, indicating that metabolic remodeling is both a consequence of neuronal hyperexcitability and a modifiable contributor to seizure severity. Flies fed an ALA-supplemented diet appeared slightly lighter and may have consumed less food than flies fed a regular diet (Supplementary Figure S1). However, this modest potential difference in food intake is unlikely affect the interpretation of our results.

### Broad Metabolic Reprogramming in *para*^*Shu*^ Mutants: Alterations in Energy Metabolism and Redox Homeostasis

Broad metabolic alterations were observed in *para*^*Shu*^ mutants. Lower levels of succinate and fumarate suggest that the TCA cycle is disrupted at the succinate–fumarate-malate branch (Fig. 4B). Notably, the 51% reduction in fumarate levels indicates that the activity of succinate dehydrogenase (SDH), which catalyzes the oxidation of succinate to fumarate, is severely impaired. SDH is unique in that it functions in both the TCA cycle and the mitochondrial electron transport chain as Complex II, transferring electrons to ubiquinone. Consequently, reduced SDH activity limits electron flow through the respiratory chain, compromises mitochondrial energy production, and may shift cellular metabolism toward greater reliance on glycolysis. Consistent with this interpretation, the accumulation of pyruvate and lactate in *para*^*Shu*^ mutants reflects a metabolic shift toward glycolysis (Fig. 4A), reminiscent of the Warburg effect observed under conditions of mitochondrial stress or elevated energy demand [44]. Similar increases in glycolytic activity, reflected by increased lactate levels, have been reported in patients with epilepsy and in rodent models of the disorder [10].

We also observed that several PPP intermediates were significantly reduced in *para*^*Shu*^ mutants. These include fructose 6-phosphate, 6-phosphogluconate, and sedoheptulose 7-phosphate (Fig. 4C). These differences suggest that PPP activity decreased, which would reduce the production of NADPH—a cofactor essential for maintaining redox homeostasis. Levels of other metabolites involved in redox regulation and the oxidative stress response were also altered. Notably, levels of the NAD⁺ precursor nicotinamide riboside (FC = 0.529, FDR = 0.00179) and nicotinic acid adenine dinucleotide (FC = 0.70, FDR = 0.00369)—a critical redox cofactor—were significantly reduced. This depletion suggests that either NAD⁺ turnover was increased or NAD⁺ biosynthesis was impaired; either could compromise the redox balance and cellular energy status. NAD^+^ depletion is a well-established phenotype in numerous models of epilepsy [45–47]. Furthermore, Liu et al. (2017) showed that NAD^+^ can suppress epileptogenesis in mice, directly linking NAD^+^ metabolism with occurrence of seizure [48]. The accumulation of homocysteine, a sulfur-containing amino acid that promotes oxidative stress (FC = 1.358, FDR = 0.025338), provides further evidence of redox dysregulation in *para*^*Shu*^ mutants.

The *para*^*Shu*^ mutation also caused significant reductions in levels of several amino acids (Table 1) that are essential for neurotransmission, energy metabolism, nitrogen homeostasis, and redox balance. For example, b-alanine, a non-proteinogenic amino acid, functions as a neuromodulator and co-agonist of GABA and glycine receptors, thereby contributing to inhibitory signaling [49]. Glutamine plays a central role in the glutamate-glutamine cycle, replenishing pools of both excitatory and inhibitory neurotransmitters [50]. Glycine serves as a co-agonist at NMDA receptors and as an inhibitory neurotransmitter, and it also contributes to one-carbon metabolism and glutathione synthesis. Proline supports redox regulation and stress responses in mitochondria, whereas arginine is a precursor of nitric oxide and polyamines, which influence synaptic signaling and cell growth. The depletion of these amino acids likely reflects a metabolic shift away from anabolic support, antioxidant defense, and neurotransmitter buffering; such changes could exacerbate neuronal hyperexcitability and reduce stress resilience.

The *para*^*Shu*^-induced reorganization of the tryptophan metabolic network (Fig. 5) is of particular interest. It is possible that a shift away from serotonin synthesis reduces neuromodulation by this neurotransmitter. Given that reduced serotonin signaling has been associated with increased seizure susceptibility [51], the observed decrease in serotonin is likely to contribute to the seizure phenotypes observed in *para*^*Shu*^ mutants. Downregulation of the kynurenine pathway intermediate 3-hydroxykynurenine can be neuroprotective, as it limits oxidative stress and excitotoxicity by reducing conversion to the neurotoxic metabolite quinolinic acid [52, 53]. However, this shift may also impair NAD⁺ synthesis, which would have adverse effects on energy metabolism. In addition, elevations in the kynurenine pathway metabolite xanthurenate are associated with altered glutamatergic signaling and metabolic dysfunction, and this could promote neuronal hyperexcitability. In contrast, the indole pathway metabolite indolepropionate is a potent antioxidant, and its increase has been associated with neuroprotection and improved metabolic health. Collectively, these findings about differences in levels in metabolites in the tryptophan pathway suggest that it may be their relative balance that critically influences neuronal excitability and systemic homeostasis. Notably, a recent study of plasma samples from 18 pediatric patients with epilepsy and 11 age-matched healthy controls demonstrated that alterations in tryptophan metabolism are a hallmark of epilepsy [9]. This underscores the commonalities in metabolic changes in epileptic patients and hyperexcitable *Drosophila* VGSC mutants, and supports the notion that tryptophan metabolism will be a good target in developing therapies for sodium channelopathies and seizure disorders.

Collectively, our findings indicate that the *para*^*Shu*^ mutation induces a profound metabolic shift characterized by impaired carbohydrate metabolism, increased reliance on glycolysis, disrupted mitochondrial function, and altered regulation of amino acid metabolism and redox homeostasis. This metabolic reprogramming likely contributes to the severity of the hyperexcitable neural phenotype in *para*^*Shu*^ mutants, underscoring a bidirectional relationship between genetically driven neuronal hyperexcitability and metabolic dysfunction. Future functional analyses—such as respirometry assays to assess mitochondrial activity and direct quantification of NAD^+^ derivatives and activities of NAD^+^-utilizing enzymes—will be required to validate the metabolic alterations identified in this study and would substantially strengthen mechanistic conclusions.

### Possible Involvement of Gut Microbiota in the Effects of *para*^*Shu*^ Mutation and Dietary ALA Supplementation

*One notable finding in this* metabolomic analysis is that the *para*^*Shu*^ mutation leads to significant alterations in metabolites of microbial origin. This observation strengthens our previous proposal that bacterial metabolites or microbiota-driven host responses exacerbate behavioral hyperexcitability, based on our earlier finding that depletion of commensal gut bacteria markedly attenuates the seizure phenotype of *para*^*Shu*^ mutants [54]. In particular, levels of SCFAs, propionic acid and butyric acid, were elevated 2.7-fold and 1.3-fold, respectively in *para*^*Shu*^ mutants (Fig. 7A, B). These SCFAs are well-established products of bacterial fermentation of dietary substrates, including carbohydrates and amino acids [55], and they can enter the systemic circulation to influence host physiology, including brain function [56]. Although SCFAs often exert beneficial effects, consistent with our finding, elevated propionate levels have been linked to pro-convulsant activity and altered neurotransmission in both animal models and humans [57, 58]. Our study also revealed substantial alterations in microbial tryptophan metabolites. Levels of tryptamine and indolepropionate were increased by 1.7-fold and 1.2-fold, respectively, whereas levels of tryptophol were decreased by 0.81-fold. Together, these results suggest that the *para*^*Shu*^ mutation alters the gut environment, thereby modifying bacterial metabolism or community composition and leading to changes in bioactive metabolites that influence seizure susceptibility.

ALA supplementation reversed many *para*^*Shu*^-induced metabolic changes, including normalization of propionate and butyrate levels. This effect may reflect modulation of the composition and/or metabolic activity of the microbiota, as dietary ω−3 fatty acids have been shown to alter microbiota profiles and SCFA production [59, 60]. The observation that bacterial depletion, ALA supplementation, and GstS1 suppression each reduce seizure severity suggests that these interventions act through partially overlapping mechanisms to buffer the effects of microbiota-derived pro-seizure metabolites. Specifically, ALA may counteract elevated SCFA levels by altering microbial fermentation patterns or host lipid signaling, whereas suppression of the *Drosophila* ortholog of mammalian prostaglandin D synthase (GstS1) likely influences prostaglandin-dependent inflammatory pathways downstream of microbiota-host interactions. A role for inflammation in modulating seizure severity in *para*^*Shu*^ mutants is further supported by several metabolite changes observed in this study. Notably, levels of N-methylnicotinamide (NMeNAM) were inversely correlated with seizure severity: NMeNAM was among the most significantly decreased metabolites in *para*^*Shu*^ mutants and was restored by ALA supplementation (Fig. 3). NMeNAM has been reported to exert anti-inflammatory effects, including activation of a COX-2–prostacyclin pathway and attenuation of inflammatory signaling in some models, with accompanying reductions in oxidative stress [61]. Moreover, our recent study demonstrated that global activation of Nrf2 produces effects that parallel those of dietary ALA supplementation, *GstS1* knockdown, and bacterial depletion [54]. Because Nrf2 signaling is known to regulate inflammatory tone, these findings collectively support a mechanistic link between neuronal hyperexcitability, metabolic state, and inflammatory response.

Together, our results support a model in which the gut microbiota contribute to the severity of the *para*^*Shu*^ seizure phenotype by producing SCFAs, tryptophan derivatives, and other metabolites that influence neuronal excitability and inflammatory tone. Our findings further suggest that interventions that modify the gut-host metabolic interface—through microbiota depletion, dietary fatty acid supplementation, or targeting prostaglandin synthesis—can attenuate seizure severity. Our preliminary 16 S rRNA gene amplicon sequencing indicates that the gut microbiota of *para*^*Shu*^ flies is dominated by *Lactobacillus* and *Acetobacter* species [54].To gain mechanistic insight into functional interactions between the gut microbiota and the seizure-prone *para*^*Shu*^ nervous system, it will be important to determine how both the composition and metabolic properties of these bacterial communities are influenced by the *para*^*Shu*^ mutation and by dietary ALA supplementation. Future studies using germ-free flies and targeted manipulation of the microbiota will also be essential for identifying the specific bacterial taxa and metabolites that mediate these effects. In addition, we plan to conduct head- and gut-specific metabolomic analyses, as such profiling is expected to provide valuable mechanistic insight into both global and tissue-specific effects of the *para*^*Shu*^ mutation and ALA supplementation.

## Conclusion

This study demonstrates that a seizure-causing gain-of-function mutation in the *Drosophila* VGSC gene induces broad and coordinated metabolic alterations encompassing energy metabolism, redox balance, amino acid and nucleotide pathways, and metabolites of microbial origin. The partial normalization of these disturbances by dietary ALA highlights the sensitivity of hyperexcitable neural states to metabolic and dietary modulation. Together, our findings establish a mechanistic link between genetically driven neuronal hyperexcitability, metabolic stress, inflammation, and gut–host interactions, and identify metabolic pathways that represent promising, testable targets for future studies aimed at mechanism-based interventions in epilepsy.

## Supplementary Information

Below is the link to the electronic supplementary material.

Supplementary Material 1

Supplementary Material 2

Supplementary Material 3

Supplementary Material 4

## Data Availability

The datasets generated and analyzed during the current study are available from the corresponding author upon reasonable request.

## Abbreviations

ALA: α-linolenic acid
GC-MS: Gas chromatography-mass spectrometry
MCFA: Medium-chain fatty acid
NAD^+^: Nicotinamide adenine dinucleotide
LC-MS: Liquid chromatography-mass spectrometry
LA: Linoleic acid
PPP: Pentose phosphate pathway
SMEI: Severe myoclonic epilepsy of infancy
SCFA: Short-chain fatty acid
TCA: Tricarboxylic acid
VGSC: Voltage-gated sodium channel
