# Supplementary material for: Metabolic Alterations Induced by a Seizure-Causing Sodium Channel Mutation and their Partial Normalization by Dietary α-Linolenic Acid in Drosophila
Source: Neurochem Res. 2026 Jan 20;51(1):51. doi: 10.1007/s11064-026-04673-2 (PMC12819498; doi:10.1007/s11064-026-04673-2)
Supplement: Supplementary file 1 — Supplementary Material 1 [file 11064_2026_4673_MOESM1_ESM.pdf]

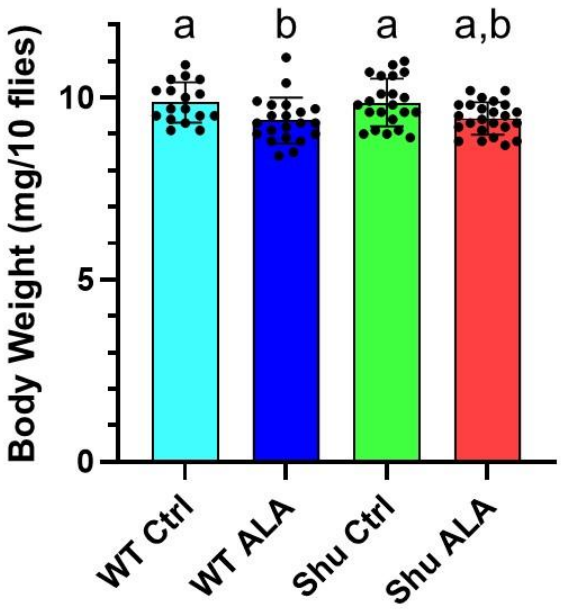

Figure S1. Body weight of wild-type flies and *para*^{Shu} mutants with or without dietary ALA supplementation.

Wild-type +/*Y* males were crossed with either wild-type females or *para*^{Shu}/*FM7* females to generate +/+ control females or heterozygous *para*^{Shu}/+ females, respectively. These *para*^{Shu}/+ heterozygotes and +/+ controls were reared on either a standard diet or a diet supplemented with 0.05% (w/v, 1.8 mM) ALA until adulthood. Virgin females (+/+ or *para*^{Shu}/+) were collected within six hours of eclosion and transferred to vials containing a standard diet (10–20 flies per vial). Flies were weighted at one day of age, and body weight was measured in groups of 10 flies. Data are shown for WT-Ctrl (cyan, N = 17), WT-ALA (navy, N = 21), Shu-Ctrl (green, N = 22), and Shu-ALA (red, N = 24), as mean ± SD. Different lowercase letters (a, b) above the bars indicate statistically significant differences among groups; groups sharing a letter are not significantly different.
