# Supplementary material for: Metabolic Alterations Induced by a Seizure-Causing Sodium Channel Mutation and their Partial Normalization by Dietary α-Linolenic Acid in Drosophila
Source: Neurochem Res. 2026 Jan 20;51(1):51. doi: 10.1007/s11064-026-04673-2 (PMC12819498; doi:10.1007/s11064-026-04673-2)
Supplement: Supplementary file 3 — Supplementary Material 3 [file 11064_2026_4673_MOESM3_ESM.docx]

|  | *Metabolite* |
| --- | --- |
| 1 | 2-Hydroxybutyrate |
| 2 | 2-Hydroxyglutarate |
| 3 | 2-Oxoadipate |
| 4 | 3-Hydroxyanthranilic acid |
| 5 | 3-Hydroxykynurenine |
| 6 | 3-Hydroxypropionate |
| 7 | 3-Phosphoglycerate |
| 8 | 6-Phosphogluconate |
| 9 | Aconitate |
| 10 | Adenine |
| 11 | Adenosine |
| 12 | Adonitol |
| 13 | Alanine |
| 14 | alpha-Keto-beta-Methylvalerate (KMV) |
| 15 | alpha-Ketoglutarate |
| 16 | alpha-Ketoisocaproate (KIC) |
| 17 | alpha-Ketoisovalerate (KIV) |
| 18 | Aminoadipate |
| 19 | Arachidate |
| 20 | Asparagine |
| 21 | Aspartate |
| 22 | beta-Alanine |
| 23 | beta-Hydroxy beta-Methylbutyric acid (HMB) |
| 24 | beta-Hydroxybutyrate (3-Hydroxybutyrate) |
| 25 | Cadaverine |
| 26 | Cholesterol |
| 27 | Citrate |
| 28 | Citrulline |
| 29 | Creatinine |
| 30 | Cysteine |
| 31 | Cytosine |
| 32 | Decanoate |
| 33 | Dihydroxyacetone phosphate (DHAP) |
| 34 | Dihydroxyphenylalanine (DOPA) |
| 35 | Docosanoate |
| 36 | Dopamine |
| 37 | Erythrose |
| 38 | Fructose |
| 39 | Fructose 6-phosphate |
| 40 | Fumarate |
| 41 | Gamma-aminobutyrate (GABA) |
| 42 | Gluconate |
| 43 | Glucose |
| 44 | Glucose 6-phosphate |
| 45 | Glutamate |
| 46 | Glutamine |
| 47 | Glyceraldehyde-3-Phosphate |
| 48 | Glycerate |
| 49 | Glycerol |
| 50 | Glycerol-3-Phosphate |
| 51 | Glycine |
| 52 | Guanine |
| 53 | Guanosine |
| 54 | Heptadecanoate |
| 55 | Heptanoic acid |
| 56 | Histamine |
| 57 | Histidine |
| 58 | Homocysteine |
| 59 | Homoserine |
| 60 | Hypotaurine |
| 61 | Hypoxanthine |
| 62 | Indoleacetate |
| 63 | Indolepropionate |
| 64 | Inosine |
| 65 | Inositol |
| 66 | Isocitrate |
| 67 | Isoleucine |
| 68 | Itaconate |
| 69 | Kynurenate |
| 70 | Kynurenine |
| 71 | Lactate |
| 72 | Laurate |
| 73 | Leucine |
| 74 | Linoleate |
| 75 | Linolenate |
| 76 | Lysine |
| 77 | Malate |
| 78 | Malonate |
| 79 | Mandelate |
| 80 | Mannose |
| 81 | Methionine |
| 82 | Mevalonate |
| 83 | Myristate |
| 84 | N-Acetylaspartate |
| 85 | N-Acetylmethionine |
| 86 | Niacin |
| 87 | Nicotinurate |
| 88 | Nonanoate |
| 89 | Oleate |
| 90 | O-Phosphoethanolamine |
| 91 | Ornithine |
| 92 | Orotate |
| 93 | Palmitate |
| 94 | Pantothenate |
| 95 | Pentadecanoate |
| 96 | Phenylalanine |
| 97 | Phosphoenolpyruvate |
| 98 | Pimelate |
| 99 | Proline |
| 100 | Pterin |
| 101 | Pyridoxal (PL) |
| 102 | Pyruvate |
| 103 | Ribose |
| 104 | Ribose 5-phosphate |
| 105 | Ribulose 5-phosphate |
| 106 | Sedoheptulose 7-phosphate |
| 107 | Serine |
| 108 | Serotonin |
| 109 | Spermidine |
| 110 | Stearate |
| 111 | Succinate |
| 112 | Taurine |
| 113 | Threonine |
| 114 | Thymine |
| 115 | Tridecanoate |
| 116 | Tryptamine |
| 117 | Tryptophan |
| 118 | Tryptophol |
| 119 | Tyrosine |
| 120 | Undecanoate |
| 121 | Uracil |
| 122 | Urea |
| 123 | Uridine |
| 124 | Valine |
| 125 | Xanthine |
| 126 | Xanthurenate |
| 127 | Xylose |
| 128 | 2,3 cyclic AMP |
| 129 | 2,3 cyclic CMP |
| 130 | 4-Hydroxy-2-nonenal |
| 131 | Acetylcholine |
| 132 | Acetyl-CoA |
| 133 | ADP |
| 134 | AMP |
| 135 | Arginine |
| 136 | ATP |
| 137 | cAMP |
| 138 | CDP |
| 139 | cGMP |
| 140 | CMP |
| 141 | CTP |
| 142 | Cytidine |
| 143 | dAMP |
| 144 | dCMP |
| 145 | dGDP |
| 146 | dGTP |
| 147 | FAD |
| 148 | GDP |
| 149 | GMP |
| 150 | GSH |
| 151 | GSSG |
| 152 | GTP |
| 153 | IMP |
| 154 | MeNAM (N-methylnicotinamide) |
| 155 | NAAD (Nicotinic acid adenine dinucleotide) |
| 156 | NAD+ |
| 157 | NADP+ |
| 158 | NAR (Nicotinic acid riboside) |
| 159 | N-Me-2PY (Nudifloramide) |
| 160 | NMN (Nicotinamide ribotide) |
| 161 | NR (Nicotinamide riboside) |
| 162 | PAP |
| 163 | Propionyl-CoA |
| 164 | Thymidine |
| 165 | TMP |
| 166 | UDP |
| 167 | UMP |
| 168 | Xanthosine |
| 169 | XMP |
| 170 | Acetic acid |
| 171 | Propionic acid |
| 172 | Butyric acid |

Supplementary Table 1. List of metabolites quantified in the untargeted metabolomic analysis used to evaluate the effects of the *para^Shu^* mutation and dietary ALA supplementation.
