# Supplementary material for: Metabolic Alterations Induced by a Seizure-Causing Sodium Channel Mutation and their Partial Normalization by Dietary α-Linolenic Acid in Drosophila
Source: Neurochem Res. 2026 Jan 20;51(1):51. doi: 10.1007/s11064-026-04673-2 (PMC12819498; doi:10.1007/s11064-026-04673-2)
Supplement: Supplementary file 4 — Supplementary Material 4 [file 11064_2026_4673_MOESM4_ESM.docx]

| *Metabolite* | *Metabolic pathway* | *F-value* | *P-value* | *FDR* | *Tukey's HSD* |
| --- | --- | --- | --- | --- | --- |
| Linolenate | Fatty Acid Metabolism | 666.34 | 3.3508e-20 | 5.7634e-18 | Shu-Ctrl-Shu-ALA; WT-ALA-Shu-ALA; WT-Ctrl-Shu-ALA; WT-ALA-Shu-Ctrl; WT-Ctrl-WT-ALA |
| Xanthurenate | Tryptophan Metabolism | 103.85 | 2.2944e-12 | 1.9731e-10 | Shu-Ctrl-Shu-ALA; WT-ALA-Shu-ALA; WT-Ctrl-Shu-ALA; WT-ALA-Shu-Ctrl; WT-Ctrl-Shu-Ctrl |
| Propionic acid | Fatty Acid Metabolism | 78.752 | 2.9909e-11 | 1.7148e-09 | Shu-Ctrl-Shu-ALA; WT-ALA-Shu-ALA; WT-Ctrl-Shu-ALA; WT-ALA-Shu-Ctrl; WT-Ctrl-Shu-Ctrl |
| Linoleate | Fatty Acid Metabolism | 60.852 | 3.1093e-10 | 1.337e-08 | Shu-Ctrl-Shu-ALA; WT-Ctrl-Shu-ALA; WT-ALA-Shu-Ctrl; WT-Ctrl-WT-ALA |
| Inositol | Inositol Metabolism | 42.998 | 6.585e-09 | 2.2652e-07 | Shu-Ctrl-Shu-ALA; WT-ALA-Shu-ALA; WT-Ctrl-Shu-ALA; WT-ALA-Shu-Ctrl; WT-Ctrl-Shu-Ctrl |
| cGMP | Nucleotide Metabolism | 29.177 | 1.6709e-07 | 4.6752e-06 | Shu-Ctrl-Shu-ALA; WT-ALA-Shu-ALA; WT-Ctrl-Shu-ALA; WT-ALA-Shu-Ctrl; WT-Ctrl-Shu-Ctrl |
| Tryptamine | Tryptophan Metabolism | 28.71 | 1.9027e-07 | 4.6752e-06 | WT-ALA-Shu-ALA; WT-Ctrl-Shu-ALA; WT-ALA-Shu-Ctrl; WT-Ctrl-Shu-Ctrl |
| Succinate | TCA Cycle | 26.967 | 3.1357e-07 | 6.7417e-06 | Shu-Ctrl-Shu-ALA; WT-ALA-Shu-ALA; WT-Ctrl-Shu-ALA; WT-ALA-Shu-Ctrl; WT-Ctrl-Shu-Ctrl |
| 3-Hydroxyanthranilic acid | Tryptophan Metabolism | 24.947 | 5.7858e-07 | 1.0416e-05 | WT-ALA-Shu-ALA; WT-Ctrl-Shu-ALA; WT-ALA-Shu-Ctrl; WT-Ctrl-Shu-Ctrl |
| Spermidine | Polyamine Metabolism | 24.801 | 6.056e-07 | 1.0416e-05 | WT-ALA-Shu-ALA; WT-Ctrl-Shu-ALA; WT-ALA-Shu-Ctrl; WT-Ctrl-Shu-Ctrl |
| XMP | Nucleotide Metabolism | 23.305 | 9.7961e-07 | 1.5317e-05 | WT-ALA-Shu-ALA; WT-Ctrl-Shu-ALA; WT-ALA-Shu-Ctrl; WT-Ctrl-Shu-Ctrl |
| NR (Nicotinamide riboside) | Nicotinate and Nicotinamide Metabolism | 22.275 | 1.3838e-06 | 1.9835e-05 | WT-ALA-Shu-ALA; WT-Ctrl-Shu-ALA; WT-Ctrl-Shu-Ctrl; WT-Ctrl-WT-ALA |
| Laurate | Fatty Acid Metabolism | 19.426 | 3.8434e-06 | 4.7336e-05 | Shu-Ctrl-Shu-ALA; WT-ALA-Shu-ALA; WT-Ctrl-Shu-ALA; WT-ALA-Shu-Ctrl; WT-Ctrl-Shu-Ctrl |
| N-Me-2PY (Nudifloramide) | Miscellaneous | 19.419 | 3.853e-06 | 4.7336e-05 | WT-ALA-Shu-ALA; WT-Ctrl-Shu-ALA; WT-ALA-Shu-Ctrl; WT-Ctrl-Shu-Ctrl |
| Sedoheptulose 7-phosphate | Glycolysis and Pentose Phosphate Pathway | 18.967 | 4.5773e-06 | 4.7823e-05 | WT-ALA-Shu-ALA; WT-Ctrl-Shu-ALA; WT-ALA-Shu-Ctrl; WT-Ctrl-Shu-Ctrl |
| Glycine | Amino Acid Metabolism | 18.964 | 4.5828e-06 | 4.7823e-05 | Shu-Ctrl-Shu-ALA; WT-Ctrl-Shu-ALA; WT-ALA-Shu-Ctrl; WT-Ctrl-Shu-Ctrl |
| Fructose 6-phosphate | Glycolysis and Pentose Phosphate Pathway | 18.883 | 4.7266e-06 | 4.7823e-05 | WT-ALA-Shu-ALA; WT-Ctrl-Shu-ALA; WT-ALA-Shu-Ctrl; WT-Ctrl-Shu-Ctrl |
| 6-Phosphogluconate | Glycolysis and Pentose Phosphate Pathway | 18.448 | 5.5974e-06 | 5.3487e-05 | Shu-Ctrl-Shu-ALA; WT-ALA-Shu-Ctrl; WT-Ctrl-Shu-Ctrl; WT-Ctrl-WT-ALA |
| Glucose 6-phosphate | Glycolysis and Pentose Phosphate Pathway | 17.233 | 9.1148e-06 | 8.2513e-05 | WT-ALA-Shu-ALA; WT-Ctrl-Shu-ALA; WT-ALA-Shu-Ctrl; WT-Ctrl-Shu-Ctrl |
| Pyridoxal (PL) | Vitamin Metabolism | 16.461 | 1.2586e-05 | 0.00010824 | Shu-Ctrl-Shu-ALA; WT-ALA-Shu-ALA; WT-Ctrl-Shu-ALA |
| CDP | Nucleotide Metabolism | 14.1 | 3.6186e-05 | 0.00029638 | WT-ALA-Shu-ALA; WT-Ctrl-Shu-ALA; WT-ALA-Shu-Ctrl; WT-Ctrl-Shu-Ctrl |
| Stearate | Fatty Acid Metabolism | 12.876 | 6.5569e-05 | 0.00051263 | Shu-Ctrl-Shu-ALA; WT-ALA-Shu-Ctrl; WT-Ctrl-Shu-Ctrl |
| NAR (Nicotinic acid riboside) | Nicotinate and Nicotinamide Metabolism | 12.548 | 7.7381e-05 | 0.00057868 | WT-ALA-Shu-ALA; WT-Ctrl-Shu-ALA; WT-ALA-Shu-Ctrl |
| Pterin | Pterin Metabolism | 12.017 | 0.0001017 | 0.00072887 | Shu-Ctrl-Shu-ALA; WT-Ctrl-Shu-ALA; WT-ALA-Shu-Ctrl; WT-Ctrl-WT-ALA |
| Xanthosine | Purine Metabolism | 11.769 | 0.00011588 | 0.00079728 | WT-ALA-Shu-ALA; WT-Ctrl-Shu-ALA; WT-ALA-Shu-Ctrl; WT-Ctrl-Shu-Ctrl |
| Indolepropionate | Tryptophan Metabolism | 11.486 | 0.00013479 | 0.00089169 | WT-ALA-Shu-ALA; WT-Ctrl-Shu-ALA; WT-Ctrl-Shu-Ctrl |
| Histamine | Amino Acid Metabolism | 10.609 | 0.00021822 | 0.0013901 | Shu-Ctrl-Shu-ALA; WT-ALA-Shu-Ctrl; WT-Ctrl-Shu-Ctrl |
| Heptanoic acid | Fatty Acid Metabolism | 10.123 | 0.00028815 | 0.0017701 | WT-ALA-Shu-ALA; WT-Ctrl-Shu-ALA; WT-ALA-Shu-Ctrl; WT-Ctrl-Shu-Ctrl |
| NAAD (Nicotinic acid adenine dinucleotide) | Nicotinate and Nicotinamide Metabolism | 9.8072 | 0.00034642 | 0.0020547 | WT-ALA-Shu-ALA; WT-Ctrl-Shu-ALA; WT-Ctrl-Shu-Ctrl |
| Serotonin | Tryptophan Metabolism | 9.3675 | 0.00045039 | 0.0025823 | WT-Ctrl-Shu-ALA; WT-ALA-Shu-Ctrl; WT-Ctrl-Shu-Ctrl |
| GSH | Glutathione Metabolism | 8.5842 | 0.00073146 | 0.0040509 | WT-ALA-Shu-ALA; WT-Ctrl-Shu-ALA; WT-ALA-Shu-Ctrl; WT-Ctrl-Shu-Ctrl |
| UMP | Nucleotide Metabolism | 8.5371 | 0.00075365 | 0.0040509 | WT-ALA-Shu-Ctrl; WT-Ctrl-Shu-Ctrl |
| NMN (Nicotinamide ribotide) | Nicotinate and Nicotinamide Metabolism | 8.3747 | 0.0008361 | 0.0043578 | Shu-Ctrl-Shu-ALA; WT-ALA-Shu-ALA; WT-Ctrl-Shu-ALA |
| Pantothenate | Vitamin Metabolism | 8.1192 | 0.00098649 | 0.0049905 | WT-ALA-Shu-Ctrl; WT-Ctrl-Shu-Ctrl |
| Proline | Amino Acid Metabolism | 7.3021 | 0.0017058 | 0.0083828 | Shu-Ctrl-Shu-ALA; WT-ALA-Shu-Ctrl; WT-Ctrl-Shu-Ctrl |
| MeNAM (N-methylnicotinamide) | Nicotinate and Nicotinamide Metabolism | 7.1593 | 0.0018829 | 0.0089959 | WT-ALA-Shu-Ctrl; WT-Ctrl-Shu-Ctrl |
| 3-Hydroxypropionate | Fatty Acid Metabolism | 7.0267 | 0.0020654 | 0.0096014 | Shu-Ctrl-Shu-ALA; WT-ALA-Shu-Ctrl |
| Butyric acid | Fatty Acid Metabolism | 6.9257 | 0.0022176 | 0.010038 | Shu-Ctrl-Shu-ALA; WT-ALA-Shu-Ctrl; WT-Ctrl-Shu-Ctrl |
| dAMP | Nucleotide Metabolism | 6.7911 | 0.0024397 | 0.010563 | Shu-Ctrl-Shu-ALA; WT-ALA-Shu-Ctrl; WT-Ctrl-Shu-Ctrl |
| Arginine | Amino Acid Metabolism | 6.7814 | 0.0024565 | 0.010563 | WT-Ctrl-Shu-ALA; WT-ALA-Shu-Ctrl; WT-Ctrl-Shu-Ctrl |
| CMP | Nucleotide Metabolism | 6.4507 | 0.0031181 | 0.013081 | WT-ALA-Shu-Ctrl; WT-Ctrl-Shu-Ctrl |
| Arachidate | Fatty Acid Metabolism | 6.1242 | 0.0039675 | 0.016248 | WT-ALA-Shu-Ctrl; WT-Ctrl-WT-ALA |
| Tryptophan | Tryptophan Metabolism | 5.8379 | 0.0049238 | 0.019695 | WT-ALA-Shu-ALA; WT-Ctrl-Shu-ALA |
| Thymine | Nucleotide Metabolism | 5.7503 | 0.0052645 | 0.020579 | WT-ALA-Shu-Ctrl; WT-Ctrl-Shu-Ctrl |
| Pyruvate | Glycolysis and Pentose Phosphate Pathway | 5.6004 | 0.0059094 | 0.022446 | Shu-Ctrl-Shu-ALA; WT-ALA-Shu-Ctrl; WT-Ctrl-Shu-Ctrl |
| Palmitate | Fatty Acid Metabolism | 5.5801 | 0.0060031 | 0.022446 | Shu-Ctrl-Shu-ALA; WT-Ctrl-Shu-ALA |
| dCMP | Nucleotide Metabolism | 5.5393 | 0.0061965 | 0.022677 | Shu-Ctrl-Shu-ALA; WT-ALA-Shu-Ctrl |
| beta-Alanine | Amino Acid Metabolism | 5.4103 | 0.0068545 | 0.024418 | Shu-Ctrl-Shu-ALA; WT-ALA-Shu-Ctrl; WT-Ctrl-Shu-Ctrl |
| Cytosine | Nucleotide Metabolism | 5.3916 | 0.0069562 | 0.024418 | WT-ALA-Shu-ALA; WT-Ctrl-Shu-ALA |
| Gamma-aminobutyrate (GABA) | Amino Acid Metabolism | 5.2866 | 0.0075578 | 0.025999 | WT-Ctrl-Shu-ALA; WT-Ctrl-Shu-Ctrl |
| Homocysteine | Amino Acid Metabolism | 5.2563 | 0.0077418 | 0.02611 | WT-ALA-Shu-ALA; WT-ALA-Shu-Ctrl |
| Fumarate | TCA Cycle | 5.2309 | 0.0079001 | 0.026131 | WT-ALA-Shu-Ctrl; WT-Ctrl-Shu-Ctrl |
| AMP | Nucleotide Metabolism | 5.172 | 0.0082804 | 0.026872 | WT-ALA-Shu-ALA; WT-Ctrl-Shu-ALA |
| Pentadecanoate | Fatty Acid Metabolism | 5.1441 | 0.0084676 | 0.026971 | WT-ALA-Shu-ALA; WT-Ctrl-Shu-ALA |
| N-Acetylmethionine | Methionine Metabolism | 5.0921 | 0.0088285 | 0.027374 | Shu-Ctrl-Shu-ALA; WT-Ctrl-Shu-ALA |
| Thymidine | Nucleotide Metabolism | 5.0804 | 0.0089123 | 0.027374 | WT-ALA-Shu-Ctrl; WT-Ctrl-Shu-Ctrl |
| Cytidine | Nucleotide Metabolism | 5.0491 | 0.0091403 | 0.027581 | WT-Ctrl-Shu-ALA |
| beta-Hydroxybutyrate (3-Hydroxybutyrate) | Fatty Acid Metabolism | 4.998 | 0.0095257 | 0.027945 | WT-ALA-Shu-Ctrl |
| UDP | Nucleotide Metabolism | 4.9902 | 0.0095858 | 0.027945 | WT-ALA-Shu-ALA; WT-Ctrl-Shu-ALA |
| Adenosine | Nucleotide Metabolism | 4.5988 | 0.01323 | 0.037333 | WT-ALA-Shu-ALA; WT-Ctrl-Shu-ALA |
| 3-Hydroxykynurenine | Tryptophan Metabolism | 4.5979 | 0.01324 | 0.037333 | WT-Ctrl-Shu-Ctrl |
| Cadaverine | Polyamine Metabolism | 4.5613 | 0.013652 | 0.037872 | WT-ALA-Shu-Ctrl; WT-Ctrl-Shu-Ctrl |
| ATP | Nucleotide Metabolism | 4.4956 | 0.014426 | 0.039386 | WT-ALA-Shu-ALA; WT-Ctrl-Shu-ALA |
| CTP | Nucleotide Metabolism | 4.3143 | 0.016825 | 0.045217 | WT-ALA-Shu-ALA; WT-Ctrl-Shu-ALA |
| Ornithine | Amino Acid Metabolism | 4.2099 | 0.0184 | 0.048689 | Shu-Ctrl-Shu-ALA; WT-ALA-Shu-ALA |
| IMP | Nucleotide Metabolism | 4.1645 | 0.019134 | 0.04972 | WT-ALA-Shu-ALA |
| alpha-Ketoglutarate | TCA Cycle | 4.148 | 0.01941 | 0.04972 | WT-ALA-Shu-Ctrl; WT-Ctrl-Shu-Ctrl |
| dGTP | Nucleotide Metabolism | 4.1334 | 0.019657 | 0.04972 | WT-ALA-Shu-ALA |

Supplementary Table 2. Metabolites showing significant differences in abundance among the four experimental groups (WT-Ctrl, WT-ALA, Shu-Ctrl, and Shu-ALA) based on one-way ANOVA followed by Tukey’s Honestly Significant Difference (HSD) test.
